# Improvement of cognitive control and stabilization of affect by prefrontal transcranial direct current stimulation (tDCS)

**DOI:** 10.1038/s41598-019-43234-2

**Published:** 2019-05-01

**Authors:** Ariane Wiegand, Anja Sommer, Vanessa Nieratschker, Christian Plewnia

**Affiliations:** Department of Psychiatry and Psychotherapy, University of Tübingen, Calwerstrasse 14, 72076 Tübingen, Germany

**Keywords:** Cognitive control, Human behaviour

## Abstract

Cognitive control of information processing is an essential prerequisite of human behavior. Particularly, focusing attention in the face of failure poses a common challenge. Previous work has demonstrated that transcranial direct current stimulation (tDCS) of the dorsolateral prefrontal cortex (dlPFC) can improve cognitive control in a challenging and repeatedly frustrating task. In a randomized, sham-controlled, crossover design 22 healthy, male participants performed an adaptive 2-back version of the Paced Auditory Serial Addition Task (PASAT), parallel to anodal or sham tDCS over the left dlPFC and the return electrode on the right upper arm. Before and after the 2-back PASAT, the affective state was assessed by means of the Positive and Negative Affective Schedule (PANAS). We observed an interaction between stimulation condition and task performance driven by an increase in performance with anodal tDCS and no improvement with sham stimulation. In addition, after the 2-back PASAT we found a higher positive and a trend towards lower negative affect with anodal as compared to sham tDCS. Our data support and extend previous results showing improved processing speed under anodal stimulation associated with a reduced task-induced negative affect indicating an improvement of cognitive control. Further studies will investigate long-term effects and clinical applicability.

## Introduction

Coping with the complexity and challenges of our daily life requires cognitive flexibility and control of our emotions and behavior. Executive functions like planning ability, response inhibition and working memory are essential to act in a goal-directed manner and to successfully master intricate situations. Cognitive control over attention is particularly important to simply focus on task-relevant information and to not get distracted by external or internal emotional stimuli^1^. Deficits in cognitive control are often involved in psychopathology of psychiatric diseases^2,3^. For instance, patients suffering from depression show an enhanced attention and an increased memory for negative emotional content^4^. This negativity bias is discussed to be caused by an impaired cognitive control over the regulation of affect as they get more easily distracted by negative stimuli^5^. On a neural basis, depression is associated with a decreased activity of the dorsolateral prefrontal cortex (dlPFC)^6,7^. As shown in several lesion and neuroimaging studies the underlying neural mechanisms of executive functions seem to involve the dlPFC as well^8–10^ and deficient cognitive control in depressive patients is associated with a hypoactivity in this brain region^11,12^.

Over the past years this association between cognitive control and dlPFC activity has also been investigated by several neurostimulation studies^13–15^. Transcranial direct current stimulation (tDCS) is a well-established and non-invasive technique to induce targeted modulation of cortical activity. It causes subthreshold alterations of the resting membrane potential and, subsequently, induces transient changes in cortical excitability^16^. Typically, anodal stimulation leads to an increase in excitability, while cathodal stimulation decreases it^17^. Several studies have been performed to examine the impact of tDCS on cognitive processes^18,19^. With regard to executive functions, anodal stimulation of the dlPFC has been associated with better working memory performances^20^, improved cognitive control^21^ and enhanced planning abilities^22^. Further, it has been shown that anodal tDCS over the left dlPFC improves impaired cognitive control in patients suffering from depression^15^, whereas cathodal stimulation of the left dlPFC in healthy subjects elicits deficits in cognitive control over negative stimuli^23^. Therefore, tDCS potentially is a promising approach to support conventional therapies for psychiatric disorders.

The present study is investigating whether 20 min of anodal tDCS over the left dlPFC affects performance in a potentially stressful and frustrating task. Participants were administered an adaptive and more challenging 2-back version of the Paced Auditory Serial Addition Task (PASAT). Previous data provided evidence that anodal dlPFC stimulation during PASAT performance improves processing speed, presumably by enhancing cognitive control on negative and distracting self-referential processes and thus suppressing task-related negative affect^24^. In line with these findings, we hypothesize to find improved processing speed and a stabilization of affect with concurrent anodal tDCS. By enhancing the activity of the dlPFC, we expect improved control on distracting changes in affect elicited by the PASAT and facilitate goal-directed behavior.

## Results

### Study Sample

Participants were randomly assigned to the order of stimulation they received during the two experimental sessions, i.e. whether their first condition was the stimulation session and the second condition the sham stimulation session (n = 11) or vice versa (n = 11). The two resulting groups showed no significant differences with respect to age, years of education, body mass index, math performance in school and their subjective estimate of their mathematical skills. Table 1 shows the demographic data in more detail.

**Table 1:** Demographic data.

	Stimulation order		*t*	*p*
	Verum/sham	Sham/verum		
	Mean (SD)	Mean (SD)		
Age [years]	24.0 (3.2)	23.2 (2.8)	0.64	0.53
Years of Education [years]	16.7 (3.5)	17.1 (3.3)	−0.25	0.80
Body mass index [kg/m^2^]	22.92 (1.78)	22.48 (2.11)	0.59	0.56
Math performance (school)*	10.1 (3.2)	10.5 (3.0)	−0.27	0.79
Math performance (subjective)**	3.2 (1.5)	2.7 (1.3)	0.74	0.47

The two groups according to stimulation order did not show any differences according to the collected data.

*According to the German academic grading system (15-point scale with 15 = very good, 0 = very bad).

**Estimates were assessed on a 7-point Likert scale (1 = very good, 7 = very bad).

### Task Performance (PASAT)

**Figure 1:**
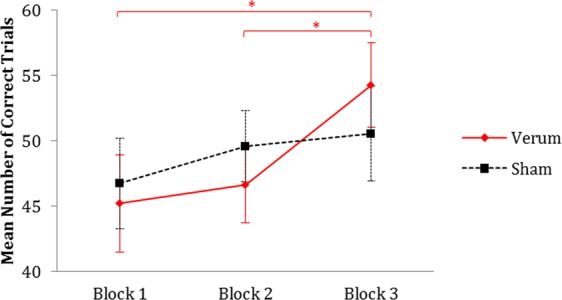
Mean numbers of correct trials with regard to stimulation. In an ANOVA the interaction of *block*_one,two,three_ and *stimulation*_verum,sham_ was significant, driven by an enhanced learning effect under verum stimulation. Error bars depict standard errors of the mean (n = 22).

The total number of trials for each participant was variable, because the inter-stimulus interval (ISI) was adapted according to the participants’ performance while the time each task block lasted remained unchanged. Thus, the adaptive design prevented significant differences in accuracy between participants. The relative frequency of errors in the PASAT did not differ between verum and sham stimulation, *t*(21) = 0.21, *p* = 0.84. Task performance was measured with respect to the number of correct trials in each task block of the PASAT. Figure 1 depicts the number of correct trials in each block for sham and anodal stimulation. An ANOVA with the repeated-measure factors *stimulation*_verum,sham_ and *block*_one,two,three_ yielded a significant main effect of *block*_one,two,three_, *F*(2,42) = 8.13, *p* = 0.001, η_p_^2^ = 0.28, indicating a learning effect. The main effect of *stimulation*_verum,sham_ was not significant, *F*(1,21) < 1, *p* = 0.94. However, a significant interaction between *block*_one,two,three_ and *stimulation*_verum,sham_ emerged, *F*(2,42) = 3.61, *p* = 0.036, η_p_^2^ = 0.15. Follow-up paired t-tests, with a Bonferroni corrected threshold for significance $(p < \frac{\alpha }{6}=0.008\bar{3})$, detected a significant increase in correct trials from task block 1 to task block 3 during the stimulation session, *t*(21) = −3.97, *p* < 0.001, whereas this increase in correct trials was not significant during the sham stimulation session, *t*(21) = −1.70, *p* = 0.10. The increase in correct trials from task block 1 to task block 2 was neither significant for the verum stimulation session, *t*(21) = −0.72, *p* = 0.48, nor for the sham stimulation session, *t*(21) = −1.44, *p* = 0.17. The increase in correct trials from task block 2 to task block 3 was significant during the verum stimulation session, *t*(21) = −3.28, *p* = 0.004, but not during the sham stimulation session, *t*(21) = −0.56, *p* = 0.58. This suggests that anodal stimulation increases the learning effect.

### Effect of Concurrent tDCS on Task-induced Mood Changes

**Figure 2:**
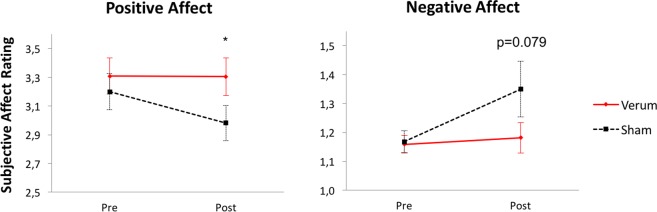
Changes in positive and negative affect with regard to stimulation. The three-way interaction of *stimulation*_verum,sham_, *time*_pre,post_ and *affect*_positve,negative_ was significant suggesting changes in affect in response to the task according to stimulation condition. Error bars depict standard errors of the mean (n = 22).

There was a significant correlation between the changes in positive and negative affect (rho = −0.37, p = 0.013), indicating the two do not evolve independently. Affective states before and after the PASAT were analyzed in an ANOVA with the within-subject factors *stimulation*_verum,sham_, *time*_pre,post_ and *affect*_positve,negative_. The three-way interaction of *stimulation*_verum,sham_, *time*_pre,post_ and *affect*_positve,negative_ was significant, *F*(1,21) = 4.90, *p* = 0.038, η_p_^2^ = 0.19. Follow-up paired t-tests detected trends for a decrease in positive affect from pre- to post-task condition, *t*(21) = 1.95, *p* = 0.065, and for an increase in negative affect, *t*(21) = −1.88, *p* = 0.074, under sham stimulation. In contrast, no significant changes in affect were detected during verum stimulation, neither for positive, *t*(21) = 0.04, *p* = 0.97, nor for negative affect, *t*(21) = −0.68, *p* = 0.50. Of note, the positive affect immediately after the PASAT was significantly higher in the verum compared to the sham stimulation session, *t*(21) = 2.33, *p* = 0.030. A trend emerged for lower negative affect after verum compared to sham stimulation, *t*(21) = −1.85, *p* = 0.079. Figure 2 depicts the positive and negative affect rating scores before and after task completion with respect to stimulation condition.

### Correlation Between Mood Changes and Task Performance

To examine whether there was a relationship between affect and task performance, changes in affect (PANAS_post_ – PANAS_pre_) during the first session were correlated with the increase in the number of correct trials (block_3_ – block_1_). Neither for positive, rho = 0.22, *p* = 0.33, nor for the negative affect, rho = −0.20, *p* = 0.37, a significant rank correlation coefficient emerged.

### tDCS Adverse Effects

At the end of both sessions participants’ adverse tDCS sensations were examined^25^. No significant differences were found between sham and verum stimulation session. Mainly, subjects felt slight tingling at the side of the electrode. Table 2 illustrates the adverse effects of tDCS in more detail.

**Table 2:** tDCS adverse effects.

Sensation	Sham tDCS	Anodal tDCS	*t*	*P*
	Mean (SD)	Mean (SD)		
Tingling at the site of the electrode	1.73 (0.98)	2.23 (1.23)	−1.67	0.11
Tingling elsewhere in the area of the head	1.09 (0.29)	1.23 (0.61)	−1.14	0.27
Exhaustion	1.05 (0.21)	1.05 (0.21)	0.00	1.00
Slight itching	1.55 (0.86)	1.82 (1.05)	−0.95	0.36
Headache	1.00 (0.00)	1.00 (000)	—	—
Nausea	1.00 (0.00)	1.00 (0.00)	—	—

Adverse sensations were assessed on a 5-point Likert scale. No significant differences between sham and anodal stimulation condition were detected (n = 22).

## Discussion

The present study aimed at adding evidence that anodal tDCS over the left dlPFC can improve cognitive control in terms of effective information processing under challenging, repeatedly frustrating conditions. We found that, in line with our previous results^24^, activity enhancing stimulation during task completion improved performance gains and was associated with a concomitant stabilization of affect. These data support the notion that anodal tDCS can exert beneficial effects on cognitive control of distractive negative information^15^ and may be harnessed for a more targeted treatment of cognitive control deficits in psychiatric disorders.

Our findings open two different but congruent perspectives on the modulatory effects of tDCS, namely on cognition and emotion. On the one hand, stimulation parallel to task performance improved goal-directed information processing, on the other hand, it prevented a deterioration in affect in response to the task. Even though the entanglement of these domains is common knowledge and essential for successful behavior, current brain stimulation research has predominantly focused on one or the other and little is known about the relevance of their mutual interactions. For instance, a convincing line of evidence exists for the efficacy of prefrontal brain stimulation as an antidepressive treatment^26–28^. In parallel, stimulation of this area has been shown to support performance and training of various executive functions^29^ like, for instance, planning ability^22^, working memory^30^, or improved problem solving^31^. However, some studies have addressed effects of tDCS on the interaction of emotion and cognition by demonstrating faster responses when inhibiting a habitual response to happy compared to sad facial expressions^32^ or by counteracting an attentional bias for emotional stimuli in depressive patients^15^. Integrating these findings and knowledge on the neural mechanisms of depression^11^ it has been suggested that the enhancement of prefrontal activity by transcranial brain stimulation can improve executive functioning including deficient inhibition on limbic brain structures^21,33^. In line with this concept, anodal tDCS to the left dlPFC can facilitate performance gains in a task requiring cognitive control of negative affect. Therefore, the present data provide further evidence for the involvement of the dlPFC in cognitive control^9,10^ and confirm its malleability by tDCS.

It is important to note that Pope *et al*.^34^ demonstrated that 2 mA anodal tDCS to the left dlPFC can improve performance in the Paced Auditory Serial Subtraction Task (PASST). Markedly, they did not find effects on the PASAT. They argue, that tDCS particularly improves cognitive performance when tasks are difficult. However, in their study, a non-adaptive and thus less difficult PASAT was used. Thus, the results are consistent with our previous study using a more challenging adaptive 1-back PASAT^24^ and the current data involving an even more difficult adaptive 2-back PASAT. However, since subjective ratings on attention and mental fatigue did not differ between stimulation and sham condition the authors speculate that the enhancement of performance is due to improved executive functioning or cognitive capacity. This is plausible but affective changes that might have been relevant, were not assessed. Another difference is the use of 1 mA stimulation intensity in the present study whereas Pope *et al*.^34^ and several other studies applied 2 mA. Considering the often non-linear interactions between stimulation intensity and behavioral effects^35^, the efficacy of both stimulation intensities while using extracephalic return electrodes demonstrates the robustness of the effect.

Importantly, our conceptual replication of the prior results^24^ with a more challenging 2-back version of the PASAT strengthens the notion of a mutual improvement of performance and affect by means of enhancing prefrontal brain activity. However, the specific relationship between these two aspects of improvement remains unresolved. Theoretically, the modulation of affect and task performance could occur independently. Nevertheless, in the light of the intricate interaction of emotion and cognition in the brain^36,37^, it appears rather unlikely that this stabilization of affect under challenging conditions could represent a mere epiphenomenon occurring independently from the concurrently improved performance. Therefore, it is reasonable to assume that focused information processing was improved by tDCS-enhanced cognitive control as reflected in enhanced processing efficacy and less occupation with negative thoughts. However, the lack of significance of the correlations between changes in affect and task performance does not support this notion but can probably be explained by a too small sample size and an insufficient precision of the PANAS.

Of note, beneficial effects of prefrontal activation with tDCS on the inhibition of distracting information is not limited to emotional content but most likely occurs in a range of diverse processes enabling focusing attention and goal-directed behavior. Several studies demonstrate that an attentional shift from external as well as self-related distractors towards task-relevant information can be facilitated by tDCS. Metuki *et al*.^31^ for example observed that participants with lower approach motivation did benefit more from anodal tDCS over the left dlPFC in a solution recognition task compared to participants with higher approach motivation. Since it can be assumed that decreased motivation positively correlates with the chance of being distracted, this might also be in line with the hypothesis that distracting information can be inhibited by tDCS. Furthermore, interindividual variability of the distractive nature of a stimulus must be considered. For instance, Sarkar *et al*.^38^ demonstrated opposite effects of tDCS on an arithmetic decision task with regard to mathematics anxiety. Whereas stimulation improved task performance and decreased cortisol levels in individuals with high mathematics anxiety, reactions were impaired in individuals with low mathematics anxiety and no decrease in cortisol levels was observed. As outlined above, this is in accordance with the assumption that tDCS reduces negative distracting information when present. Taken together, in context of previous work our results support the idea of the dlPFC being involved in top-down regulation of task-oriented information processing and suppression of task-irrelevant information. This capacity appears to be supported by concomitant tDCS.

From a clinical perspective, increased attention to negative stimuli, unbalanced emotion processing and higher levels of self-referential negative affect are characteristic of depression^4^. The administration of the PASAT has been shown to evoke negative emotional states by frustration and causes increased self-referential thinking and rumination in healthy subjects^39,40^. Therefore, the PASAT appears to challenge neurocognitive networks, particularly the prefrontal cortex^41^, that are critically involved in cognitive control^42^ and the pathophysiology of depression^5^. By functionally targeting these networks in patients with deficient cognitive control, a combination of PASAT training and anodal tDCS to the prefrontal cortex could be able to leverage therapeutic efficacy^43^.

Several limitations should be considered. First, the affective state was only measured before and after the PASAT. Therefore, respective information during the PASAT is missing and should be the subject of future studies. Second, the use of self-report questionnaires might not be sufficiently precise to detect small or latent mood changes. Especially in the negative affect there might be a floor effect. Therefore, neurophysiological signatures of the effects should be obtained in future studies. Third, the single blind design of the study might have influenced the dependent variables, however, to avoid this all instructions were given following a fixed detailed script. Fourth, it could be assumed that the mood deteriorations we observed during sham stimulation occur because a worse task performance and therefore more frequent error feedback. However, due to the adaptive task design of the 2-back PASAT, a better performance is linked with a faster stimulus presentation, which in turn leads to more mistakes. This is indicated by similar percentages of error feedback between the conditions.

In summary, our data provide further evidence that anodal electrical stimulation of the left dlPFC stabilizes affect under cognitively challenging conditions and shifts attention from distracting stimuli towards task-relevant processing and, thereby, facilitates goal-directed behavior. This underlines the essential role of the dlPFC in executive functioning and efficient cognitive control. Particularly in the clinical context, prefrontal tDCS in combination with training of cognitive control might be a potential approach to complement conventional therapies for psychiatric disorders but more research is needed to decipher the effectiveness of stimulation protocols and its biological basis.

## Materials and Methods

### Participants

According to a power analysis performed by G*Power (Version 3.1) based on the effect size of previous results^24^ (ANOVA, repeated measures, within factors: η_p_² = 0.18 (f = 0.47), α = 0.05, power = 80%, nonsphericity correction ε = 0.20) a sample size of N = 17 would be large enough to detect differences between conditions. To correct for a possible overestimation, we increased the sample size by 1/3^rd^ to 22 subjects.

Hence, 22 healthy, male participants aged between 18–30 years took part in this experiment. Screening excluded participants with a history of any mental or neurological illness, dyscalculia, metallic foreign particles around the head, a cardiac pacemaker, and the usage of anti-psychotic, hypnotic, or sedative medications. The participants (mean age: 23.6 years, SD = 3.0; mean education: 16.9 years, SD = 3.3) were all right-handed according to the Edinburgh Handedness Inventory (LI = 96.61, SD = 8.29)^44^, non-smoking and German native speakers. All participants gave written informed consent to the experimental procedure prior to study conduction. The study was performed in accordance with the Declaration of Helsinki and approved by the University of Tübingen local ethics committee.

### Adaptive 2-Back Paced Auditory Serial Addition Task (PASAT)

The Paced Auditory Serial Addition Task (PASAT) has originally been designed to assess cognitive impairments after traumatic brain injury^45^ but has later been developed into a computer-based version to induce stress under standardized conditions to assess neuropsychological functioning^46^. In the task of this study, single digits ranging from 1 to 9 were presented via headphones. The participants were asked to add the currently named number to the number named before the previous one and type in the correct result by pressing the correspondingly labeled keyboard button. This more difficult 2-back version of the PASAT was developed to sufficiently challenge cognitive control in the healthy young participants. For each trial, they received visual feedback simultaneously with the onset of the next stimulus. Specifically, the screen flashed green for a correct answer and red for an incorrect, late or missed answer. The inter-stimulus interval (ISI) between digit presentations was initially set to 3 s but was decreased by 0.1 s if the participant gave four consecutive correct answers and increased by 0.1 s after four consecutive false answers. Therefore, the total number of trials was variable and dependent upon the participant’s performance. The PASAT consisted of 16 practice trials followed by three task blocks lasting for 5 min each, which were separated by breaks of 30 s. The total number of correct responses was used as dependent variable. To increase the participants’ ambition, they were informed that the three best participants receive vouchers valued 15 €, 10 € and 5 €, respectively.

### Positive and Negative Affect Schedule (PANAS)

The Positive and Negative Affect Schedule (PANAS) is a self-report to determine the participants’ current affective states^47^. In this study, the German version of the PANAS was used^48^. It comprises 20 items which were rated on a five-point Likert scale ranging from 1 ‘not at all’ (in German: ‘gar nicht’) to 5 ‘very much’ (in German: ‘äußerst’) to measure positive or negative affect (10 items each). To assess changes in any of the affective states during the experimental procedure participants completed the PANAS twice throughout each session: before starting the PASAT (pre) and immediately after they completed the PASAT (post).

### Transcranial Direct Current Stimulation (tDCS)

A direct current of 1 mA was generated by a portable, battery-driven stimulator (NeuroConn GmbH, Illmenau, GER) and applied via a pair of 5 × 7 cm electrodes. The anodal electrode was encased in saline-soaked sponges and additionally covered with conductive paste (Ten20®, Weaver and Company, Aurora, CO) and placed over the left dlPFC at F3 according to the international 10–20 system of electrode placement^49^. The cathodal reference electrode was fixated on the right upper arm over the deltoid muscle to prevent any opposite polarization of other brain regions^15,50^. The stimulation onset was 2 min before the participants started the PASAT and the current was faded in for 5 s. During the anodal stimulation session, a continuous current of 1 mA was delivered for 20 min until task completion and then faded out for another 5 s, whereas during the sham condition the current was only administered for 30 s before it was ramped down for 5 s. The impedance was controlled by the device and ranged below 10 kΩ.

### Experimental Procedure

The study followed a single-blind, sham-controlled, within-subject design. Each participant attended two sessions, which differed in the type of stimulation (sham/verum) they received during task completion. The order of stimulation was counterbalanced across participants and the second session followed 7 days after the first one. To minimize variability, all sessions started at 2 pm. Before the beginning of the first session subjects signed the informed consent and participated in a brief screening including the Symptom-Checklist-90-Revised (SCL-90-R) to determine the global distress level (GSI)^51^ to ensure the inclusion criteria. From here on the experimental procedure was the same for both sessions except for the administered stimulation. To avoid influences by the single-blind design of the study on the dependent variables, all instructions were given following a fixed detailed script. The participants were equipped with tDCS electrodes and, in the context of another research question, a peripheral venous catheter was placed. Then the first PANAS was administered. While receiving tDCS (sham or verum) they were exposed to the 2-back PASAT. Immediately after task completion the PANAS was administered for a second time. Finally, subjects were asked about their tDCS adverse sensations.

### Statistical analysis

All statistical calculations were performed with SPSS (IBM SPSS Statistics 22.0; Ehningen; Germany). As a measure of task performance the number of correct trials was used instead of the ISI, as the number of correct trials is a more sensitive and direct measurement, because the ISI did just change after each run of four consecutive correct or four consecutive false trials. Mean numbers of correct trials in each of the three task blocks were extracted from the adaptive 2-back PASAT as measure of task performance. To detect any stimulation effects these values were subjected to an ANOVA with the within-subjects factors *stimulation*_verum,sham_ and *block*_one,two,three_. Results were considered significant when p < 0.05. Post-hoc pairwise comparisons were performed after significant interactions.

For the PANAS, means were calculated for the 10 items each comprising positive and negative affect. The scores of the two PANAS questionnaires administered before and immediately after the PASAT were subjected to an ANOVA with the within-subjects factors *stimulation*_verum,sham_, *time*_pre,post_ and *affect*_positve,negative_. Besides, the PANAS questionnaire administered immediately after task completion was examined in more detail by subjecting the mean scores for positive and negative affect to an ANOVA with the within-subject factors *stimulation*_verum,sham_ and *affect*_positve,negative_. Paired t-tests were used to investigate item-specific differences according to stimulation condition.

To test for a correlation between affect changes (PANAS_POST_ – PANAS_PRE_) and PASAT improvement (#correct trials_Block 3_ - #correct trials_Block 1_) Spearman’s Rho nonparametric rank correlation coefficient for Likert scale differences was calculated. Only data from the first session of each participant were included in this analysis to ensure independence of the measures.

## Data Availability

The datasets generated and analyzed during the current study are available from the corresponding author on reasonable request.

## References

[1] Ochsner KN, Gross JJ (2005). The cognitive control of emotion. Trends in Cognitive Sciences.

[2] Goschke T (2014). Dysfunctions of decision-making and cognitive control as transdiagnostic mechanisms of mental disorders: advances, gaps, and needs in current research. Int J Methods Psychiatr Res.

[3] Ottowitz WE, Dougherty DD, Savage CR (2002). The neural network basis for abnormalities of attention and executive function in major depressive disorder: implications for application of the medical disease model to psychiatric disorders. Harv Rev Psychiatry.

[4] Bradley BP, Mogg K, Williams R (1995). Implicit and explicit memory for emotion-congruent information in clinical depression and anxiety. Behaviour Research and Therapy.

[5] Fales CL (2008). Altered emotional interference processing in affective and cognitive-control brain circuitry in major depression. Biol Psychiatry.

[6] Baxter LR (1989). Reduction of prefrontal cortex glucose metabolism common to three types of depression. Arch Gen Psychiatry.

[7] Galynker II (1998). Hypofrontality and negative symptoms in major depressive disorder. J Nucl Med.

[8] Barbey AK, Colom R, Grafman J (2013). Dorsolateral prefrontal contributions to human intelligence. Neuropsychologia.

[9] MacDonald AW, Cohen JD, Stenger VA, Carter CS (2000). Dissociating the role of the dorsolateral prefrontal and anterior cingulate cortex in cognitive control. Science.

[10] Ridderinkhof KR, van den Wildenberg WP, Segalowitz SJ, Carter CS (2004). Neurocognitive mechanisms of cognitive control: the role of prefrontal cortex in action selection, response inhibition, performance monitoring, and reward-based learning. Brain Cogn.

[11] Disner SG, Beevers CG, Haigh EA, Beck AT (2011). Neural mechanisms of the cognitive model of depression. Nat Rev Neurosci.

[12] Koenigs M, Grafman J (2009). The functional neuroanatomy of depression: distinct roles for ventromedial and dorsolateral prefrontal cortex. Behav Brain Res.

[13] Moreno ML (2015). Effects of acute transcranial direct current stimulation in hot and cold working memory tasks in healthy and depressed subjects. Neurosci Lett.

[14] Shin YI, Foerster A, Nitsche MA (2015). Reprint of: Transcranial direct current stimulation (tDCS) - Application in neuropsychology. Neuropsychologia.

[15] Wolkenstein L, Plewnia C (2013). Amelioration of cognitive control in depression by transcranial direct current stimulation. Biol Psychiatry.

[16] Nitsche MA (2008). Transcranial direct current stimulation: State of the art 2008. Brain Stimul.

[17] Nitsche MA, Paulus W (2000). Excitability changes induced in the human motor cortex by weak transcranial direct current stimulation. J Physiol.

[18] Kuo MF, Nitsche MA (2012). Effects of transcranial electrical stimulation on cognition. Clin EEG Neurosci.

[19] Wassermann EM, Grafman J (2005). Recharging cognition with DC brain polarization. Trends Cogn Sci.

[20] Brunoni AR, Vanderhasselt MA (2014). Working memory improvement with non-invasive brain stimulation of the dorsolateral prefrontal cortex: a systematic review and meta-analysis. Brain Cogn.

[21] Plewnia C, Schroeder PA, Wolkenstein L (2015). Targeting the biased brain: non-invasive brain stimulation to ameliorate cognitive control. Lancet Psychiatry.

[22] Dockery CA, Hueckel-Weng R, Birbaumer N, Plewnia C (2009). Enhancement of planning ability by transcranial direct current stimulation. J Neurosci.

[23] Wolkenstein L, Zeiller M, Kanske P, Plewnia C (2014). Induction of a depression-like negativity bias by cathodal transcranial direct current stimulation. Cortex.

[24] Plewnia C, Schroeder PA, Kunze R, Faehling F, Wolkenstein L (2015). Keep calm and carry on: improved frustration tolerance and processing speed by transcranial direct current stimulation (tDCS). PLoS One.

[25] Brunoni AR (2011). A systematic review on reporting and assessment of adverse effects associated with transcranial direct current stimulation. Int J Neuropsychopharmacol.

[26] Kalu UG, Sexton CE, Loo CK, Ebmeier KP (2012). Transcranial direct current stimulation in the treatment of major depression: a meta-analysis. Psychol Med.

[27] Mutz Julian, Edgcumbe Daniel R., Brunoni Andre R., Fu Cynthia H.Y. (2018). Efficacy and acceptability of non-invasive brain stimulation for the treatment of adult unipolar and bipolar depression: A systematic review and meta-analysis of randomised sham-controlled trials. Neuroscience & Biobehavioral Reviews.

[28] Nitsche MA, Boggio PS, Fregni F, Pascual-Leone A (2009). Treatment of depression with transcranial direct current stimulation (tDCS): a review. Exp Neurol.

[29] Imburgio MJ, Orr JM (2018). Effects of prefrontal tDCS on executive function: Methodological considerations revealed by meta-analysis. Neuropsychologia.

[30] Ruf SP, Fallgatter AJ, Plewnia C (2017). Augmentation of working memory training by transcranial direct current stimulation (tDCS). Sci Rep.

[31] Metuki N, Sela T, Lavidor M (2012). Enhancing cognitive control components of insight problems solving by anodal tDCS of the left dorsolateral prefrontal cortex. Brain Stimul.

[32] Vanderhasselt MA (2013). tDCS over the left prefrontal cortex enhances cognitive control for positive affective stimuli. PLoS One.

[33] De Raedt R, Vanderhasselt MA, Baeken C (2015). Neurostimulation as an intervention for treatment resistant depression: From research on mechanisms towards targeted neurocognitive strategies. Clin Psychol Rev.

[34] Pope PA, Brenton JW, Miall RC (2015). Task-Specific Facilitation of Cognition by Anodal Transcranial Direct Current Stimulation of the Prefrontal Cortex. Cereb Cortex.

[35] Batsikadze G, Moliadze V, Paulus W, Kuo MF, Nitsche MA (2013). Partially non-linear stimulation intensity-dependent effects of direct current stimulation on motor cortex excitability in humans. J Physiol.

[36] Barbas, H. & García-Cabezas, M. Á. In *The Prefrontal Cortex as an Executive*, *Emotional*, *and Social Brain* (ed. Masataka Watanabe) 51–76 (Springer Japan, 2017).

[37] Huntsinger, J. R. & Schnall, S. In *The Oxford Handbook of Cognitive Psychology* (2013).

[38] Sarkar A, Dowker A, Cohen Kadosh R (2014). Cognitive enhancement or cognitive cost: trait-specific outcomes of brain stimulation in the case of mathematics anxiety. J Neurosci.

[39] Holdwick DJ, Wingenfeld SA (1999). The subjective experience of PASAT testing. Does the PASAT induce negative mood?. Arch Clin Neuropsychol.

[40] Tombaugh TN (2006). A comprehensive review of the Paced Auditory Serial Addition Test (PASAT). Arch Clin Neuropsychol.

[41] Audoin B (2005). Functional MRI study of PASAT in normal subjects. MAGMA.

[42] McTeague LM (2017). Identification of Common Neural Circuit Disruptions in Cognitive Control Across Psychiatric Disorders. Am J Psychiatry.

[43] Vanderhasselt MA (2015). Transcranial electric stimulation and neurocognitive training in clinically depressed patients: a pilot study of the effects on rumination. Prog Neuropsychopharmacol Biol Psychiatry.

[44] Oldfield RC (1971). The assessment and analysis of handedness: the Edinburgh inventory. Neuropsychologia.

[45] Gronwall, D. M. & Sampson, H. *The psychological effects of concussion*. (Auckland U Press, 1974).

[46] Lejuez, C., Kahler, C. W. & Brown, R. A. A modified computer version of the Paced Auditory Serial Addition Task (PASAT) as a laboratory-based stressor. *The Behavior Therapist* (2003).

[47] Watson D, Clark LA, Tellegen A (1988). Development and validation of brief measures of positive and negative affect: the PANAS scales. J Pers Soc Psychol.

[48] Krohne HW, Egloff B, Kohlmann C-W, Tausch A (1996). Untersuchungen mit einer deutschen Version der “Positive and Negative Affect Schedule”(PANAS). Diagnostica-Gottingen.

[49] Jasper HH (1958). The ten twenty electrode system of the international federation. Electroencephalography and Clinical Neurophysiology.

[50] Priori A (2008). Lie-specific involvement of dorsolateral prefrontal cortex in deception. Cereb Cortex.

[51] Derogatis, L. R. Administration, scoring, and procedures manual for the SCL-90-R. *Baltimore*, *MD: Clinical Psychometrics Research* (1977).

